# Excessive Daytime Sleepiness and Risk of Sleep Apnea: A Retrospective Analysis of Peruvian Adults

**DOI:** 10.2174/0117450179442008251205104706

**Published:** 2026-02-25

**Authors:** Víctor Juárez-Chunga, Aurora Calderón-Valentín, Doris Hancco-Huillca, Tania Betsy Rios-Tuanama, Andre Nelson Sotomayor-Serruno, Juan Morales

**Affiliations:** 1 Faculty of Health Sciences, University of Sciences and Humanities (UCH), Lima, Peru; 2 Department of Neurology, Hospital II-2 of Tarapoto, San Martín, Peru; 3 University of Sciences and Humanities (UCH), e-Health Research Center, Lima, Peru

**Keywords:** Excessive daytime sleepiness, Sleep apnea central, Sleep apnea obstructive, Peru, Apnea-hypopnea index, Epworth sleepiness scale, Central sleep apnea

## Abstract

**Introduction:**

Sleep apnea is a highly prevalent chronic disorder and a leading causal factor of excessive daytime sleepiness (EDS).

**Objectives:**

This study aimed: (1) To evaluate the presence of EDS and the type of sleep apnea in the adult population assessed; (2) to identify factors associated with the type of sleep apnea; and (3) to evaluate the ability of EDS to predict the type of sleep apnea.

**Materials and Methods:**

This study employed an observational, descriptive, and cross-sectional design, utilizing a retrospective analysis of secondary data. A total of 350 adults evaluated at a specialized sleep center in Lima, Peru, were included. EDS was assessed using the Epworth Sleepiness Scale (ESS), while the type of sleep apnea was determined through overnight polysomnography.

**Results:**

EDS was present in 84.6% (n= 296) of participants, with a predominance of ESS levels II and III. According to the polysomnographic diagnosis, 8.0% (n = 28) showed no evidence of sleep apnea, 49.1% (n = 172) were diagnosed with central sleep apnea, and 42.9% (n = 150) with obstructive sleep apnea. Male sex (OR = 5.32; 95% CI: 2.01–14.06) and symptoms, such as daytime sleepiness (OR = 3.51; 95% CI: 1.52–8.10), difficulty falling asleep (OR = 5.20; 95% CI: 2.32–11.64), awakening with sleepiness (OR = 35.11; 95% CI: 8.16–151.07), and choking sensation upon awakening (OR = 15.62; 95% CI: 2.10–116.42), were significantly associated with sleep apnea. EDS emerged as the main predictor in the decision tree model, which achieved an overall accuracy of 58.5%.

**Discussion:**

Daytime sleepiness is associated with sleep apnea, underscoring its clinical relevance. However, the model´s moderate predictive accuracy reinforces the importance of polysomnography as the gold standard diagnosis.

**Conclusion:**

EDS was found to be highly prevalent and significantly associated with both central and obstructive sleep apnea. These findings highlight the clinical importance of screening for EDS in primary care as a practical approach to identifying individuals at higher risk of sleep apnea, supporting timely referral for polysomnography.

## 1 INTRODUCTION

Sleep is a complex physiological process regulated at global, regional, and local levels through cellular and molecular mechanisms [1]. It plays a vital role in both physical and mental health by facilitating recovery, repair, and growth of the body [2, 3]. Quality sleep contributes to the optimal functioning of multiple body systems, including the neurological, cardiovascular, immune, and endocrine systems, as well as cognitive performance, alertness, emotional stability, and psychological well-being [1-3]. In healthy individuals, the recommended sleep duration is 7 to 9 hours for young and middle-aged adults, and 7 to 8 hours for older adults [4]. Sleep disturbances have a negative impact on cognitive and emotional functioning and are associated with the exacerbation of various health problems, such as metabolic disorders, cardiovascular diseases, depression, and anxiety [2, 5].

Excessive daytime sleepiness (EDS) is a significant public health concern [6]. It may result from behaviors that lead to insufficient or fragmented sleep, as well as from sleep disorders, including sleep apnea syndrome, circadian rhythm disorders, central hypersomnia disorders, medical or psychiatric conditions, and the use of certain medications [6, 7]. Prevalence estimates in the general population range from 12% to 18% [8-10]. Elevated rates have also been reported among healthcare professionals and university students, highlighting the occupational and academic relevance of this phenomenon [11, 12]. EDS has important health implications, as it has been identified as a potential risk factor for various conditions, including cardiovascular diseases, neurodegen-erative disorders, and gastrointestinal conditions [6, 13]. Additionally, it has been associated with reduced quality of life, an increased risk of accidents, and decreased work performance [14].

Sleep apnea is a chronic respiratory disorder characterized by intermittent reductions (hypopneas) or complete cessations (apneas) of airflow during sleep. It is estimated to affect approximately 10% of the general population and has been associated with a threefold increased risk of heart disease, a fourfold risk of stroke, and a sevenfold risk of motor vehicle accidents [15]. Based on the presence or absence of respiratory effort, sleep apnea is classified as either obstructive sleep apnea (OSA) or central sleep apnea (CSA) [15]. Both forms are among the most frequently associated causes of EDS. In particular, one of the main complications of OSA is EDS [16-18].

The prevalence of OSA in the general population is higher than that of CSA [19]. In recent years, OSA has increased worldwide, becoming a major public health concern. The global prevalence of EDS among patients with OSA is 40% (95% CI: 34.4–45.7) [16]. OSA affects more than 900 million adults aged 30 to 69 worldwide [18]. In Peru, the prevalence of OSA in this same age group is 53%, with moderate to severe cases accounting for 27% [20].

CSA is another condition that may present with EDS [21]. It is estimated to account for 5% to 10% of cases diagnosed in clinical settings. However, its prevalence is considerably higher among individuals with heart failure, certain neurological disorders, and those receiving high-dose opioid treatment [22]. On the other hand, OSA is a major factor associated with cardiovascular morbidity, including systemic and pulmonary hypertension, heart failure, acute coronary syndromes, atrial fibrillation, and other arrhythmias [23]. Both OSA and CSA have been linked to an increased frequency of atrial fibrillation [24].

In recent years, most epidemiological evidence has focused on OSA, leaving CSA comparatively underexplored. Moreover, although EDS is a common symptom in both OSA and CSA, its predictive value for distinguishing between apnea types has not been adequately investigated. Identifying whether EDS can serve as a practical predictor could enhance clinical screening and prioritization of polysomnographic studies in resource-limited settings.

Therefore, this study aimed to determine whether EDS can predict the type of sleep apnea in adults undergoing polysomnography. The specific objectives were: (1) to evaluate the presence of EDS and the type of sleep apnea in the adult population assessed; (2) to identify factors associated with the type of sleep apnea; and (3) to evaluate the ability of EDS to predict the type of sleep apnea.

## 2 MATERIALS AND METHODS

### 2.1 Study Type and Design

The study adopted a quantitative approach and an observational, descriptive, and cross-sectional design, with a retrospective analysis of secondary data. The use of retrospective data allowed access to a large sample evaluated under standardized clinical and diagnostic conditions.

### 2.2 Study Population and Sample

The study population consisted of adult patients evaluated at a specialized neurophysiology center in Lima, Peru. The sampling was census-based, including all patients assessed between 2020 and 2025 who met the following criteria.

Inclusion criteria were as follows: adults aged ≥18 years with a presumptive diagnosis of obstructive sleep apnea, central sleep apnea, hypopnea, or narcolepsy who completed overnight polysomnography. Exclusion criteria included incomplete clinical or polysomnographic records, missing Epworth Sleepiness Scale (ESS) data, or inconclusive diagnostic results. The final analytical sample comprised 350 complete and validated records.

### 2.3 Study Variables

#### 2.3.1 Excessive Daytime Sleepiness (EDS)

EDS was defined as the tendency of an individual to doze off or fall asleep, even briefly, at a particular moment in time [25]. The Epworth Sleepiness Scale (ESS) was used to assess this condition.

#### 2.3.2 Sleep Apnea

Sleep apnea is a disorder characterized by repeated pauses in breathing during sleep. It is classified into obstructive sleep apnea (OSA) and central sleep apnea (CSA). Apnea resulting from upper airway obstruction is known as OSA, whereas apnea caused by impaired respiratory control is referred to as CSA [26]. According to the American Academy of Sleep Medicine (AASM), apnea is defined as a ≥90% reduction in airflow from baseline during sleep, with duration criteria specified in the AASM Scoring Manual for both adult and pediatric patients.

The diagnosis of OSA is based on the presence of symptoms and either polysomnography (PSG) or home sleep apnea testing (HSAT) showing ≥5 predominantly obstructive respiratory events per hour of sleep, or PSG/HSAT showing >15 predominantly obstructive respiratory events per hour of sleep [27].

The following groups of variables were included as covariates: sociodemographic variables, oropharyngeal anatomical assessment, anthropometric variables, medical history, and health habits. All variables were collected by trained clinical personnel to minimize information bias.

#### 2.3.3 Sociodemographic Variables

The following variables were collected for each participant: sex, marital status, place of residence, occupation, and presumptive diagnosis.

#### 2.3.4 Oropharyngeal Anatomical Assessment

The Modified Mallampati Scale was applied, which classifies the visibility of oropharyngeal structures into four grades (I-IV) [28].

#### 2.3.5 Anthropometric Variables

Anthropometric assessments included weight, height, body mass index (BMI), abdominal circumference, and neck circumference.

#### 2.3.6 Medical History and Health Habits

Medical history and health habits were assessed, including the presence of arterial hypertension, diabetes mellitus, family history of diabetes, disability, night shift work, alcohol consumption, tobacco use, and engagement in physical activity.

#### 2.3.7 Clinical Findings

Sleep-related symptoms were recorded as present or absent (yes/no). These included snoring, nocturnal drowsiness, ease of falling asleep, nocturnal awakenings, daytime sleepiness, sleep episodes while driving, waking up with drowsiness, difficulties with concentration or memory, insomnia, awakening with a sensation of choking, night sweats, sleep talking, and nocturia.

### 2.4 Measurement Instruments

#### 2.4.1 Excessive Daytime Sleepiness (EDS)

To assess EDS, the Epworth Sleepiness Scale (ESS) was used. The ESS is a self-administered and internationally validated instrument [29]. It consists of 8 items that evaluate the likelihood of dozing off or falling asleep in various daily situations. Each item is scored from 0 to 3 (0 = none, 1 = slight, 2 = moderate, 3 = high), yielding a total score ranging from 0 to 24. Higher scores indicate a greater propensity for sleep. Traditionally, scores above 10 are associated with sleep disorders, while values greater than 16 reflect severe levels of sleepiness, commonly observed in patients with narcolepsy, idiopathic hypersomnia, or obstructive sleep apnea syndrome. The ESS has been validated in several countries, demonstrating adequate reliability for the self-reported assessment of daytime sleepiness [25]. In Peru, an adapted and validated version is available for the adult population, with psychometric properties comparable to those of the original version [30].

#### 2.4.2 Polysomnography Diagnosis of Sleep Apnea

The diagnosis of sleep apnea was performed using overnight polysomnography (PSG), which is considered the gold standard for detecting sleep-related breathing disorders, OSA, CSA, and sleep-related hypoventilation or hypoxemia [31, 32].

An Electroencephalograph Amplifier BW III PSG PLUS device (serial number BWII 2023-4089) was used, standardized according to the AASM diagnostic criteria. According to this institution, PSG is the reference diagnostic test for confirming OSA in adults, particularly when clinical findings suggest its presence following a comprehensive sleep evaluation [32].

### 2.5 Data Quality and Missing Data Management

Data were exported from the medical records and verified by a researcher who did not have access to the primary source. Discrepancies were resolved through chart review. Missing or inconsistent data accounted for less than 1% of all cases and were assessed individually.

### 2.6 Statistical Analysis

Numerical variables were assessed for normality using the Lilliefors test. Categorical variables were summarized using frequencies and percentages; continuous variables were summarized using medians and interquartile ranges.

Group comparisons were made with the Kruskal–Wallis test and Bonferroni-adjusted post hoc tests. Associations between categorical variables were analyzed using the Chi-square or Fisher’s exact test, as appropriate. Crude Odds Ratios (OR) and 95% confidence intervals (95% CI) were estimated.

For predictive modeling, a decision tree analysis was implemented. The model was trained with all independent variables, and cross-validation pruning was applied to avoid overfitting. The decision tree was chosen for its interpretability and clinical applicability in exploratory predictive modeling. The model achieved an overall classification accuracy of 58.5%.

All analyses were performed in RStudio 4.5.0, and a significance level of *p* < 0.05 was used.

### 2.7 Ethical Considerations

Access to the database was authorized by the authorities of the corresponding healthcare facility. Ethical principles of confidentiality, anonymity, and data protection were strictly observed according to the Declaration of Helsinki. This study was approved by a Research Ethics Committee.

## 3 RESULT

A total of 350 participants of both sexes were included in the analysis, with a median age of 55 years (Min: 34, Max: 90, Q1:48, Q3:61). A presumptive diagnosis of obstructive sleep apnea (OSA) was present in 74.3% (n = 260) of the evaluated individuals (Table **1**).

Excessive daytime sleepiness (EDS), measured by the Epworth Sleepiness Scale (ESS), and oropharyngeal features according to the modified Mallampati score, were both significantly associated with the presence of sleep apnea (*p* < 0.001) (Table **2**).

**Fig. (1):**
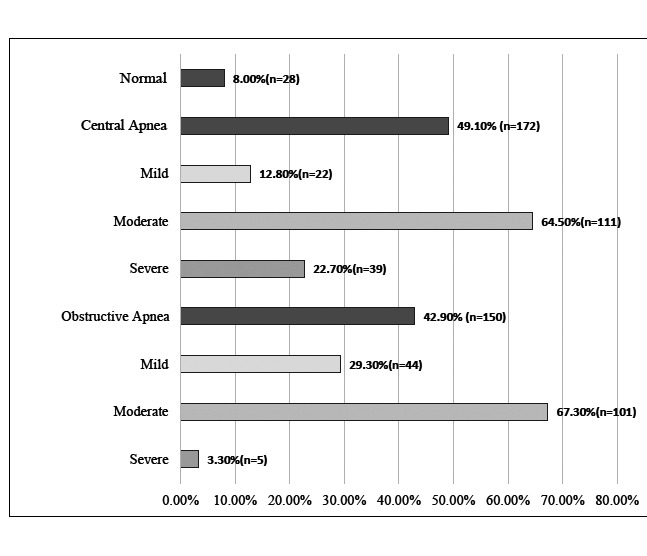
Types of Sleep Apnea.

Regarding apnea classification, 8.0% (n = 28) of participants had no evidence of sleep apnea. CSA was diagnosed in 49.1% (n = 172), of which 64.5% (n = 111) showed moderate severity. OSA was identified in 42.9% (n = 150), with 67.3% (n = 101) of cases classified as moderate (Fig. **1**).

Significant differences in age and anthropometric variables were found among the three groups (no apnea, CSA, and OSA) (Kruskal–Wallis test, *p* < 0.005). Post hoc comparisons with the Bonferroni correction indicated that participants with OSA were older and had higher body weight than those with CSA (*p* < 0.001 and *p* = 0.004, respectively). BMI, abdominal, and neck circumferences were also significantly greater in both the OSA and no-apnea groups compared to the CSA group (*p* < 0.01) (Table **3**).

Among sociodemographic variables, male sex (OR = 5.32; 95% CI: 2.01–14.06) and family history of type 2 diabetes mellitus (OR = 4.59; 95% CI: 1.07–19.75) were significantly associated with a higher likelihood of sleep apnea (Table **4**).

Clinical features significantly related to sleep apnea included EDS (OR = 3.51; 95% CI: 1.52–8.10), difficulty initiating sleep (OR = 5.20; 95% CI: 2.32–11.64), awakening with sleepiness (OR = 35.11; 95% CI: 8.16–151.07), and awakening with a choking sensation (OR = 15.62; 95% CI: 2.10–116.42) (Table **5**).

**Fig. (2):**
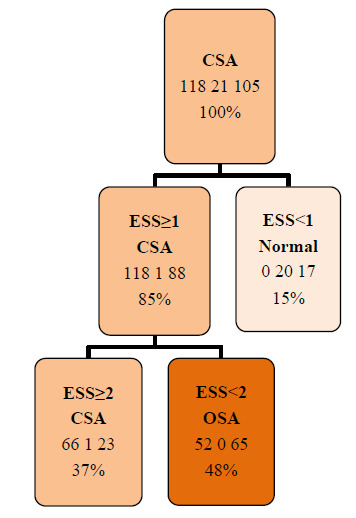
Pruned Decision Tree. CSA: Central Sleep Apnea, ESS: Epworth Sleepiness Scale, OSA: Obstructive Sleep Apnea.

Finally, the decision tree model identified EDS as the most relevant predictor for classifying sleep apnea type. The model achieved an overall accuracy of 58.5%, indicating moderate discriminative performance (Fig. **2**).

## 4 DISCUSSION

The present study aimed to evaluate the presence of EDS and the type of sleep apnea in Peruvian adults, identify associated factors, and determine the predictive value of EDS in classifying apnea type. Overall, the findings confirm that EDS is highly prevalent among adults referred for suspected sleep disorders and that it retains moderate predictive capacity for distinguishing between obstructive and central apnea.

The high frequency of EDS observed (84.6%) highlights the burden of impaired sleep regulation in this clinical population. The association between ESS scores and apnea underscores the clinical relevance of EDS as a potential indicator of sleep-disordered breathing. However, this elevated prevalence likely reflects the characteristics of the studied population rather than the general population. The finding that nearly nine out of ten individuals with apnea reported EDS reinforces the importance of daytime sleepiness as an accessible clinical marker, although its diagnostic specificity remains limited.

The ESS quantifies the severity of EDS, with scores ≥10 indicating clinically significant sleepiness [33]. Elevated ESS scores have been associated with a higher risk of OSA (OR = 1.19; 95% CI: 1.07–1.31) [34]. Despite being a simple, self-administered, and widely used instrument, its diagnostic accuracy is limited. The ESS has a high false-negative rate, reducing its value as a standalone screening tool. Reported sensitivity ranges from 0.27 to 0.72 and specificity from 0.50 to 0.76 when using an Apnea–Hypopnea Index (AHI) threshold of ≥5 [32, 35].

In the present study, 49.1% of participants were diagnosed with CSA and 42.9% with OSA, showing a slight predominance of CSA. This pattern contrasts with most reports, where OSA is consistently the most common type of sleep apnea [36, 37]. The discrepancy likely reflects the characteristics of the sample and the influence of associated comorbid conditions.

In the current research, individuals with OSA tended to be older and have higher weight and body mass indicators than those without apnea, consistent with evidence linking OSA to age, male sex, and obesity [23, 34, 38]. These associations reinforce the role of anthropometric and metabolic factors in the pathophysiology of sleep-related airway obstruction. Consistent with previous evidence, male sex emerged as a major risk factor. However, the elevated risk observed in overweight and postmenopausal women suggests that hormonal decline and body fat redistribution may also contribute to apnea susceptibility [39].

Although no personal pathological history was significantly associated with sleep apnea in this study, previous evidence has consistently linked the condition to hypertension, excess body weight, and diabetes [34]. In particular, a BMI ≥ 25 markedly increases the risk of OSA (OR = 10.75, 95% CI: 8.21–14.06), as do hypertension (OR = 5.83, 95% CI: 3.91–8.69), diabetes (OR = 2.54, 95% CI: 1.46–4.42), and hyperlipidemia (OR = 2.85, 95% CI: 1.36–5.95) [34]. Other chronic conditions, such as heart failure, chronic nasal obstruction, nasal polyps, sinusitis, and the use of sedatives or tranquilizers, have also been identified as contributing factors [39].

In this study, EDS, as measured by ESS, emerged as the most robust predictor of sleep apnea type. The decision tree achieved an overall accuracy of 58.5%, correctly identifying the apnea type in more than half of the cases. Although the ESS is not a substitute for objective diagnostic methods, such as PSG, its inclusion in the model yielded acceptable discriminative performance within this clinical cohort. The AASM does not recommend clinical tools, questionnaires, or predictive algorithms as diagnostic substitutes for OSA in adults in the absence of PSG or home sleep apnea testing [32]. Nevertheless, despite its inherent limitations [32, 35], the ESS may serve as a useful preliminary screening instrument in settings with restricted access to polysomnographic evaluation or in populations with a high pretest probability of sleep apnea.

EDS represents a cardinal symptom of OSA [40, 41]. Although CSA shares overlapping clinical manifestations, its identification is particularly relevant, as it may reflect unrecognized underlying pathologies and guide more targeted therapeutic strategies [19]. Both OSA and CSA substantially contribute to cardiovascular and pulmonary morbidity [23, 24]; therefore, their early recognition, even in the presence of nonspecific or overlapping symptoms, is essential to optimize diagnostic accuracy and treatment outcomes.

## 5 STUDY LIMITATIONS

This study has several limitations that should be considered. First, the data analyzed were obtained from a secondary source, which limits control over data quality. Second, probabilistic sampling procedures were not applied; therefore, the results cannot be generalized to the broader population. Additionally, EDS alone cannot define the type of sleep apnea; it serves only as an indicator of a possible sleep disorder. While apnea is the most common cause of EDS, other etiologies are also possible. Nevertheless, the study includes a clinically relevant and substantial sample size, which strengthens the validity of the findings. In this context, the results may be useful to guide interventions and clinical decision-making in populations with similar characteristics within healthcare settings.

## CONCLUSION

Excessive daytime sleepiness (EDS) was found to be a prevalent condition in the evaluated population. A total of 92% of patients presented with some form of sleep apnea, with central sleep apnea as the most common subtype. Patients with obstructive sleep apnea exhibited significantly higher values of age, weight, body mass index, abdominal circumference, and neck circumference. The main risk factors identified for sleep apnea included male sex, EDS, difficulty falling asleep, awakening with sleepiness, and nocturnal episodes of choking sensation. In the predictive model, EDS emerged as the strongest predictor of apnea type, suggesting its potential as an early clinical marker. In this context, the Epworth Sleepiness Scale (ESS), due to its practicality, self-administered format, and ease of use, serves as a valuable supporting tool in the initial evaluation of patients with suspected sleep apnea. Its application facilitates the early identification of cases and timely referral to specialized services; however, it does not replace polysomnography, which remains the diagnostic gold standard.

## Figures and Tables

**Table 1:** Sample characteristics.

Sample characteristics	n	%
Total	350	100
**Sex**		
Females	26	7.4
Male	324	92.6
**Age group**		
≤40	40	11.4
41-49	73	20.9
50-59	120	34.3
≥60	117	33.4
**Marital status**		
Single	23	6.6
Married	113	32.3
Cohabiting	104	29.7
Separated	102	29.1
Widowed	8	2.3
**Residence**		
Lima	287	82
Province	58	16.6
Foreign	5	1.4
**Occupation**		
Armed Forces Officer	97	27.7
Retired Armed Forces Personnel	54	15.4
Healthcare Professional	46	13.1
Homemaker	15	4.3
Assisting Professional	28	8
Student	18	5.1
Other	92	26.3
**Presumptive Diagnosis**		
Obstructive Sleep Apnea	260	74.3
Central Apnea	46	13.1
Hypopnea	41	11.7
Narcolepsy	3	0.9

**Table 2:** Epworth sleepiness scale and mallampati score and their association with sleep apnea.

Variables	Total	Normal	Apnea	p
	n (%)	n (%)	n (%)	
Total	350(100)	28(100)	172(100)	
**Daytime Sleepiness**				
Yes	296(84.6)	18(64.3)	278(86.3)	0.002
No	54(15.4)	10(35.7)	44(13.7)	
**Epworth Sleepiness Scale**				
I (0 a 5)	53(15.1)	26(92.9)	27(8.4)	<0.001
II (6-10)	160(45.7)	1(3.6)	159(49.4)	
III (11-15)	119(34.0)	1(3.6)	118(36.7)	
IV (16-20)	18(5.1)	0(0.0)	18(5.6)	
V (>20)	0(0.0)	0(0.0)	0(0.0)	
**Mallampati Scale**				
I/IV	38(10.9)	17(60.7)	21(6.5)	<0.001
II/IV	133(38.0)	4(14.3)	129(40.1)	
III/IV	142(40.6)	1(3.6)	141(43.8)	
IV/IV	37(10.6)	6(21.4)	31(9.6)	

**Table 3:** Age and anthropometric measurements by sleep apnea type.

Variables	Normal	CSA	OSA	*p*-value
	Median (Q1-Q3)	Median (Q1-Q3)	Median (Q1-Q3)	
Age	56 (50-65)	52(44.8-60)	55.6(49-65)	0.001
Body weight	91 (83-96.6)	78(72-88.5)	84.6(73.2-96)	<0.001
Height	1.70(1.62-1.74)	1.66(1.60-1.69)	1.68(1.60-1.73)	0.029
BMI	31.6(29.9-33.2)	29(26.9-30.9)	30.3(27.7-32.3)	<0.001
Waist circumference	102(99.8-109)	97.5(95-100)	99(95-104)	<0.001
Neck circumference	41.2(40-43)	39(38-41)	40(39-42)	<0.001

**Abbreviations:** CSA: Central Sleep Apnea, OSA: Obstructive Sleep Apnea.

**Table 4:** Risk factors associated with sleep apnea.

Medical History and Habits	Total		Apnea		Normal		*p*-value	OR (95% CI)
	n	%	n	%	n	%		
Total	350	100	322	100	28	100		
**Sex**								
Male	324	92.6	303	94.1	21	75	<0.001	5.32 (2.01-14.06)
Female	26	7.4	19	5.9	7	25		
**Body Mass Index**								
Normal	25	7.7	25	7.8	0	0	0.126	NA
Excess Body Weight	325	92.3	297	92.2	28	100		
**Medical History**								
Yes	57	16.3	54	16.8	3	10.7	0.405	1.68 (0.49-5.76)
No	293	83.7	268	83.2	25	89.3		
**Disability**								
Yes	11	3.1	11	4.4	0	0	0.668	NA
No	339	96.9	311	96.6	28	100		
**Night Shift Work**								
Yes	82	23.4	78	24.2	4	14.3	0.234	1.92 (0.65-5.7)
No	268	76.6	244	75.8	24	85.7		
**Arterial Hypertension**								
Yes	59	16.9	55	17.1	4	14.3	0.705	1.24 (0.41-3.7)
No	291	83.1	267	82.9	24	85.7		
**Diabetes Mellitus (DM)**								
Yes	58	16.6	56	17.4	2	7.1	0.162	2.74 (0.63-11.87)
No	292	83.4	266	82.6	26	92.9		
**Family History of DM**								
Yes	86	24.6	84	26.1	2	7.1	0.026	4.59 (1.07-19.75)
No	264	75.4	238	73.9	26	92.9		
**Alcohol Consumption**								
Yes	173	49.4	152	47.2	21	75	0.005	0.30 (0.12-0.72)
No	177	50.6	170	52.8	7	25		
**Cigarette Smoking**								
Yes	140	40	130	40.4	10	35.7	0.629	1.22 (0.55-2.72)
No	210	60	192	59.6	18	64.3		
**Physical Activity**								
Yes	150	42.9	123	38.2	27	96.4	<0.001	0.02 (0.00-0.17)
No	200	57.1	199	61.8	1	3.6		

**Table 5:** Clinical findings associated with apnea.

Clinical Findings	Total		Apnea		Normal		*p*-value	OR (95% CI)
	n	%	n	%	n	%		
Total	350	100	322	100	28	100		
**Snoring**								
Yes	301	86	278	86.3	23	82.1	0.539	1.37 (0.50-3.80)
No	49	14	44	13.7	5	17.9		
**Daytime sleepiness**								
Yes	296	84.6	278	86.3	18	64.3	0.002	3.51 (1.52-8.10)
No	54	15.4	44	13.7	10	35.7		
**Ease of falling asleep**								
No	59	16.9	46	14.3	13	46.4	<0.001	5.2 (2.32-11.64)
Yes	291	83.1	276	85.7	15	53.6		
**Sleep interruptions**								
Yes	76	21.7	73	22.7	3	10.7	0.141	2.44 (0.72-8.32)
No	274	78.3	249	77.3	25	89.3		
**Sleeping during the day**								
Yes	74	21.1	72	22.4	2	7.1	0.059	3.74 (0.87-16.15)
No	276	78.9	250	77.6	26	92.9		
**Falling asleep while driving**								
Yes	26	7.4	26	8.1	0	0	0.118	NA
No	324	92.6	296	91.9	28	100		
**Waking up feeling sleepy**								
Yes	237	67.7	235	73	2	7.1	<0.001	35.11 (8.16-151.07)
No	113	32.3	87	27	26	92.9		
**Problems with concentration or memory**								
Yes	33	9.4	33	10.25	0	0	0.075	NA
No	317	90.6	289	89.75	28	100		
Insomnia								
Yes	5	1.4	5	1.55	0	0	0.507	NA
No	345	98.6	317	98.45	28	100		
**Waking up with choking sensation**								
Yes	119	34	118	36.7	1	3.6	<0.001	15.62 (2.10-116.42)
No	231	66	204	63.4	27	96.4		
**Night sweats**								
Yes	41	11.7	41	12.7	0	0	0.044	NA
No	309	88.3	281	87.3	28	100		
**Sleep talking**								
Yes	31	8.9	29	9	2	7.1	0.739	1.29 (0.29-5.70)
No	319	91.1	293	91	26	92.9		
**Nocturia**								
Yes	149	42.6	123	38.2	26	92.9	<0.001	0.05 (0.01-0.20)
No	201	57.4	199	61.8	2	7.1		

## Data Availability

The data that support the findings of this study are available from https://doi.org/10.5281/zenodo.17173216.

## LIST OF ABBREVIATIONS

AASM: American Academy of Sleep Medicine.
AHI: Apnea-Hypopnea Index.
BMI: Body Mass Index.
CSA: Central Sleep Apnea.
EDS: Excessive Daytime Sleepiness.
ESS: Epworth Sleepiness Scale.
HSAT: Home Sleep Apnea Testing.
IQR: Interquartile Range.
NA: Not Applicable.
OR: Odds Ratio.
OSA: Obstructive Sleep Apnea.
PSG: Polysomnography.
Q1: 25th percentile.
Q3: 75th percentile.
95%CI: 95% confidence interval.

## STANDARDS OF REPORTING

STROBE guidelines were followed.
